# Ethical Stakes for Past, Present, and Prospective Tuberculosis Isolate Research Towards a Multicultural Data Sovereignty Model for Isolate Samples in Research

**DOI:** 10.1007/s11673-023-10334-8

**Published:** 2024-05-27

**Authors:** A. Anderson, M. Meher, Z. Maroof, S. Malua, C. Tahapeehi, J. Littleton, V. Arcus, J. Wade, J. Park

**Affiliations:** 1 Te Kupenga Hauora Māori, University of Auckland, Auckland, New Zealand; 2 School of Social Sciences, The University of Auckland, Auckland CBD, Auckland, 1010 New Zealand; 3 The University of Waikato, Knighton Road, Hamilton, 3240 New Zealand

**Keywords:** Indigenous data sovereignty, Tuberculosis research, Kaupapa Māori research, Isolate samples

## Abstract

Tuberculosis (TB) is a potentially fatal infectious disease that, in Aotearoa New Zealand (NZ), inequitably affects Asian, Pacific, Middle Eastern, Latin American, and African (MELAA), and Māori people. Medical research involving genome sequencing of TB samples enables more nuanced understanding of disease strains and their transmission. This could inform highly specific health interventions. However, the collection and management of TB isolate samples for research are currently informed by monocultural biomedical models often lacking key ethical considerations. Drawing on a qualitative kaupapa Māori–consistent study, this paper reports on preliminary discussions with groups of Māori, Pacific, and Afghan people in NZ, whose communities have been harmed by TB and TB stigma. Participants’ discussions highlight key concerns and meanings that ought to inform the development of guidelines and a more robust consultative process for the governance of how TB isolate samples are collected and used both retrospectively and in future research. We argue for ethical processes to be culturally nuanced and community-generated, flexible and meaningful, and situated in relation to the physical and symbolic effects of TB. We discuss the significance of Indigenous data sovereignty, rights, and kāwanatanga (governorship) in shaping a multicultural data sovereignty model.

## Background

Tuberculosis (TB) is an infectious disease that is described by the World Health Organization (WHO) as a global epidemic (WHO 2022). WHO reports that 1.6 million people died of TB in 2021, making it the second most fatal infectious disease (after COVID-19) and a WHO priority pathogen. Aotearoa New Zealand (NZ) is considered a low TB incidence country by the New Zealand Ministry of Health (Ministry of Health 2023), with a comparatively low total TB incidence rate, averaging 6.2–7 cases per 100,000 people each year between 2007 and 2019 (Institute of Environmental Science and Research (ESR) 2022). This relatively low rate masks differences characterized by ethnicity and socio-economic inequity seen across different populations in NZ: Asian 25.8/100,000; Middle Eastern, Latin American, and African (MELAA) 20.7/100,000; Pacific peoples 13.7/100,000; Māori 3.5/100,000; European or other; 0.4/100,000 (ESR 2022).

Newly available techniques including whole genome sequencing of diseases such as TB offer novel opportunities for yielding data of significant potential for health interventions. Analyses of TB pathogen isolates, the bacterial or viral organisms that grow in human sputum, could reveal whether TB recurrences represent reactivation of latent strains or infection of a different strain. This knowledge could make it possible to trace the origins of TB infection and TB transmissions within local communities and inform the basis of effective interventions and services. More nuanced understandings of TB infection could also challenge stereotyping narratives which can stigmatize groups carrying the highest rates of infection (Anderson 2008a). For example, a prolific molecular type of TB, the Rangipo strain, was named due to a patient in a large 2001 Hawkes Bay outbreak who served time in Rangipo prison (McElnay, Thornley, and Armstrong 2004). The high rates of transmission of this strain have been incorrectly ascribed to prison populations, stigmatizing those individuals who become infected with this molecular type and contributing towards deficit framing of those communities (not to mention reinforcing stigma towards incarcerated people). Recent whole genome sequencings of this strain have indicated its high transmission rates may be due to increased virulence rather than due to it originating in a prison (Gautam et al. 2017) and that the strain is likely of European origin, prompting some researchers to suggest it be renamed CS1 (“colonial strain 1”) (Mulholland et al. 2019). Further analyses of TB strains may be able to confirm this and allow the deconstruction of this and other harmful narratives.

Human pathogen-based laboratory practices, whether for research or health-response delivery, are predicated on a Western biomedical view that pathogen “isolates” are isolated entities and disconnectable from the bodies which they inhabit. This assumption means that Indigenous or other cultural views about the extraction of pathogen isolates have not been sought or taken into account. Yet for many cultures there are significant beliefs around health and bodies that can influence engagement with research or healthcare relating to biological or pathogen sample collections. For example, for many Māori (the Indigenous people of NZ) there is a tapu (sacredness) associated with physical samples taken from the body (e.g., sputum) along with data derived from these samples. Tapu has been described by Durie (1994, 8) at its simplest as situations that are “off limits” in either physical or spiritual terms.

This monocultural approach to isolate research or clinical use creates a number of questions about how data sovereignty could be maintained over pathogen samples taken from human bodies, and what culturally responsive practice could look like for pathogen-based laboratory research. Especially at a time when, under new technologies, whole genome sequencing of human pathogens is becoming routine and vast amounts of genomic data from pathogens are now being collected and stored or biobanked (Ministry of Health 2006). It is timely and important that new community-driven guidelines are established and practiced for this data. There is precedent for such developments in the fields of genomic research and lab work (Hudson, Farrar, and McLean 2016), Indigenous medicine and health systems (Boulton et al. 2014), and research with human tissue samples and cervical cancer cells (Te Whatu Ora 2023).

Further, uncertainties remain about how best to treat TB biological samples that have already been collected and stored where it may not be possible to retrospectively obtain consent. In the absence of guidelines for culturally appropriate treatment of TB pathogen samples or stored isolates obtained from samples, decisions about whether these samples should be used, how they might be used, and what research questions they should be used to answer are best made by the communities from whom they were taken and for whom the research should benefit.

To elucidate these complex issues, this research investigated understandings of culturally appropriate approaches to human-derived microbial-pathogen laboratory research by canvassing the perspectives of three community groups—Māori, Afghan, and Pacific—on the management of pathogen samples taken from human bodies and on the use of existing collections of TB samples.

Over several decades, there has been greater acknowledgement in healthcare, health research, and health data sectors of the value of community input to inform ethical and effective processes and outcomes for diverse populations (Yuan, et al. 2021). This study enquired into what communities felt were benefits and challenges of research using TB isolate samples for their respective communities. We asked participants to speak into a space governed exclusively by biomedical ethical frameworks, in which there is presently no community generated ethical oversight or input. Their discussions demonstrated that there is much at stake for people in relation to TB and isolate sample research. Specific to each of these groups were their relationship to the colonial nation of NZ and their historical relationships with medicine and research. The richness of participants’ considered contributions—their convictions, anxieties, and compassion—alone form an argument for the importance of developing culturally informed, community-generated ethical guidelines around pathogen sample collection and use in TB research. They reveal aspects of TB sample collection and custodianship that are contentious and sensitive, that call for a robust, consolidated, and informed process in order to develop appropriate guidelines.

## Methods

### Methodology

This study undertook a qualitative kaupapa Māori consistent approach. Kaupapa Māori methodology prioritizes Māori paradigms and operates under a decolonizing lens by critiquing structures of power allowing research to operate within a critical framework that avoids cultural deficit explanations (Mahuika 2008). A kaupapa Māori consistent approach aligns with the key principles of kaupapa Māori methodology yet may be led by or may include non-Māori researchers and participants (Malpas, et al. 2017). This research was led by a Māori researcher and was approved by Tōmaiora, a kaupapa Māori research group at the University of Auckland. The research included three ethnic groups: Māori, Afghan, and a Pacific group (who did not want their ethnicity reported in publications due to the stigma associated with TB). These communities were included to provide a diverse range of experiences from Indigenous, diaspora migrant, and refugee perspectives. The experiences of MELAA communities, including Afghan, are currently under-represented in New Zealand and add an additional voice to the growing body of TB literature, which to date has included qualitative research with Pacific (Ng Shiu, Park, and Kearns 2008; Littleton and Park 2009; Dunsford et al. 2011) and Asian (Anderson 2008a, b; Littleton et al. 2008) community experiences with TB.

Kaupapa Māori research gives full recognition to Māori leadership, control, cultural values, and systems (Walker, Eketone, and Gibbs 2006). To fulfil these principles, kaumātua (Māori elders) guided the research design and engagement with Māori, ensuring that Māori tikanga (cultural protocols) were observed through all meetings and research processes. Furthermore, mutual respect within the research team enabled the research to work towards hauora (well-being) for Māori and other communities involved. The model of consulting with community leaders to guide the research process was also used for Pacific and Afghan community engagement, data collection, and analysis. Researchers from each community were involved in every aspect of the research to ensure the cultural values and paradigms of each community were valued, prioritized, and embedded in the research outcomes, in alignment with kaupapa Māori principles.

### Data Collection and Analysis

The study followed a two-stage process. At stage one, leaders from each community were identified and consulted with regarding the most culturally responsive ways of engaging with their respective communities and asked who should be included as participants.

Stage two involved consultation-based data collection with members of the communities about human-pathogen research and the TB isolates in particular. Potential participants were identified from community researchers and community leaders. People were invited to participate via verbal, phone, or email invitations, then provided with information sheets about the study and consent forms. Ethics approval was granted for the study in 2018 by the University of Auckland Human Participants Ethics Committee (Ref 020830).

Different data collection methods were employed for each community based on their respective tikanga, languages, and systems. All interviews or hui (formalized meetings) were undertaken in the first language of participants (Māori, a Pacific language, Fārsī, English). Each interview began presenting basic illustration-based information about TB and isolates to participants to provide foundational contexts for understanding. Next, participants were asked questions around their experiences of TB, their perceptions of using TB isolates for research, cultural appropriateness of isolate collection, storage, and use, and who they thought should be involved in these processes.

Two hui were undertaken with Māori participants. The first hui was undertaken with kaumātua at a church in the Waikato region. The second hui was held with rangatahi (young adults) at a private residence in Auckland. Hui followed tikanga through a mihi whakatau (welcome), karakia (prayers), whakawhānaungatanga (relationship building), and sharing kai (food).

Face to face interviews were held with members across three ethnic Afghan communities: Tajik, Pashtun, and Hazara. Six interviews were held in Auckland and two in Waikato. Some of these involved gendered groups who were interviewed by a gender-matched researcher, and some were couples interviewed by the researchers.

Two focus group interviews were held with the Pacific community. The first focus group was held with men, the second with women. Both focus groups were held at a community centre in Auckland. Pacific meetings began with a prayer, and food was provided.

Interviewers were ethnically matched with interviewees (and gender matched for interviews with Afghan participants) to keep the research culturally responsive. Data was audio recorded and accompanied by handwritten notes and observations.

Audio data was transcribed and translated into English. Data was thematically analysed using a general inductive approach (Thomas 2006). Seven researchers of Māori, Pacific, Afghan, and Pākehā ethnicities individually identified codes (based on key ideas, concepts, and commonly used words and phrases) through individual repeated readings of the transcripts in the first instance. Themes were then identified from patterns and underlying similarities that emerged from the respective coding frameworks through collaborative and consensus-based discussions.

## Results

There were thirty participants in the research. Participants included a diversity of people in terms of ethnicity, gender, age, education, occupation, and time spent living in NZ (table 1). Participants ranged from sixteen to seventy years of age.

**Table 1:** Participants by ethnic communities

Ethnicity	Female	Male
Māori	7	1
Pacific	5	9
Afghan	3	5

The findings firstly describe the medical and social understandings of TB shared by participants, including the fear and stigma associated with TB in each community group. Subsequent findings report on discussions and ethical concerns raised by participants surrounding four distinct aspects of isolate collection, storage, and custodianship: 1) the values of consent and tikanga pertinent to isolate collection, 2) concerns raised associated with personal information and the privacy of samples, 3) questions over who has the right to speak for others in a community in a local governance scenario, and 4) the investment across all the groups in collective well-being and collective good, including the ways that participants conceptualized love, service, and TB research as entangled.

### Medical and Social Understandings of TB

Almost all Afghan and many Pacific participants’ understandings of TB were informed by past personal experiences with TB in their families overseas, where the person with TB was isolated and treatment often managed within the household, with serious and painful implications. The sole exception to this kind of close experiences was an Afghan interviewee who had come to NZ very young. In the group of kaumātua, there was also personal experiences with TB close to home, often in the past, some also credited a local doctor with providing information at a town hall session following a recent infection in the community. Interviewees had appreciated the doctor assuaging their fears of transmission, explaining that “it wasn’t that easy to catch,” and answering their many questions. In contrast to the other groups, TB for rangatahi was experienced more externally, passed on through stories in their whānau or via media reports.

Many participants across the communities requested and recommended more health education around TB for their communities. In interviews, participants asked many questions, and appreciated how the space to kōrero (talk) helped fill out and detail their understandings of TB in ways that information sheets and presentations without discussion time could not.

Participants expressed a broad lay understanding of TB, aligning, at times, with biomedical discourse of infections caused by “bugs,” and recognized social, contextual factors, including different occupations, poverty, and household crowding. For them all, TB was a grave matter medically, both for its severity, with many likening it to cancer or cardiovascular disease in terms of long-term impact and likelihood of death, and its transmissibility. Participants expressed a general understanding of the symptoms of TB, yet a lay understanding did not always include details of TB infectiousness and TB treatment, hence its transmissibility and severity were especially feared, and great caution and care were exercised around the “bug.” Several Afghan and Pacific participants relayed stories of TB close to home involving long, undetermined isolation periods and safety measures of using separate eating implements for the person with TB and boiling their plates and cutlery to disinfect them. One man explained that “if a guest, who had TB before, was coming to our house, we used to boil the spoon and plate for hours after he/she left. He/she might have been non-contagious, but we did not know.” In one discussion, interviewees were interested to learn that TB only spreads through breathing in an infected person’s cough, and that with a consistent drug treatment regime, isolation is only necessary for two weeks. Similarly, through conversation with researchers in the hui/focus groups, being able to visualize the “bug,” as “clever” and as “living in a greasy envelope” that is difficult to penetrate helped participants reframe it as a knowable, manageable threat.

People’s fear and anxiety of TB was also linked to the racialized, deficit framing of TB in media reports, which negatively impacted some Pacific communities socially and concerned some Afghan and Pacific participants in relation to delaying their residency or securing residence visas for NZ. One Afghan participant who had experienced non-pulmonary TB (TB infecting organs other than the lungs) earlier in life was worried and depressed coming up to his immigration chest X-Ray, fearing visa denial or deportation. A Pacific interviewee noted that one of the questions on visa applications is if you are from “a TB country.” He added “everyone has been branded, but we understand why.” Some Pacific interviewees felt that Māori were in a more privileged position as they did not have legal barriers of citizenship and migration to contend with. Some Māori participants questioned whether national TB caseloads could be linked to immigration, harkening to historical whānau and community experiences of contagion coming from overseas. Implicit in these conversations is the stigma related to TB—a core theme in our study. One group asked the researchers, with some concern, why their community was included for this study, wary of having been singled out as members of a TB-carrying community. Similarly, in another group, feedback from the broader community came that there was upset about TB-related research being carried out with this ethnic group, as this was felt to “stir the pot.” We discuss stigma further in relation to participants’ thoughts on contact tracing, personal information collected with samples, the right to speak for others, and countering stigma with love. These discussions reveal the nuanced expressions of TB stigma, affecting individuals, families, and communities, provoking blame, seclusion, isolation, stress, and anxiety at personal and collective levels. These tensions are a powerful reminder of the potential “name and shame” nature of the combined effects of media coverage and contact tracing for TB.

### Collecting Isolates: Consent and Tikanga

All participants supported the use of TB isolates to “help” cure, prevent, and manage TB. Across the individually and culturally diverse thoughts offered elaborating on this, two major points of consideration emerged: consent to collect and how collected substances were treated and managed. The handling of substances rests with how the materiality of isolates were culturally understood in each group.

Afghan and Pacific participants considered TB isolates as being distinct from the body and therefore having no cultural or religious significance if taken for research. “After all, they are bacteria!” said one Afghan participant, with a big laugh, somewhat mirroring the Pacific participant who described isolates as “only the bug,” not part of the human body.

One of the Pacific focus groups did challenge the assumption that consent was by default an individual matter. Some participants thought the decision to participate in TB research should be a family/community decision because of the shared stigma, in that families share the stigma of one member.

In discussions with Māori participants, there were greater subtleties at play in considering the tapu nature of isolates. Everybody in the group of kaumātua, and common amongst rangatahi, agreed that it was a complex question. They all agreed that there was mauri (life force/essence) in TB isolates as they had been in the human body, which is considered tapu. Kaumātua offered a cricket game analogy for how mauri animates substances:

… when a cricket ball is bowled by a cricket player in a cricket pitch the game is in play aye, so the mauri is there… every time the ball leaves the bowler’s hand the mauri is in play; when the ball is hit with the bat it’s in play. Mauri is not a body; it’s something else.

For some, the spit that carried TB isolates was significant here too, as from/of the body, though not bodily tissue or a body part itself. Conversations with Māori participants suggested that priorities shifted from context to context. One participant said organ donation was important to her, so health trumped tapu in such cases, even if she thought her whānau (family) would disagree. Two others said they would prefer pathogen isolate research to human tissue research, specifically because it was not, say, a heart or kidney. These complexities and nuances show that it would be immensely important to establish appropriate protocol and tikanga around the materiality of collecting and using isolates in research.

The suggestions offered for developing tikanga-based ethical guidelines entailed involving Māori with “well versed knowledge of tikanga Māori” (as a rangatahi put it) in the process. Kaumātua suggested inclusion of whakanoa (practices to remove tapu) and karakia when collecting samples and when leaving home to go to hospital. Further recommendations were ensuring the involvement of kaumātua and kaimahi (Māori health/lab workers) or a Māori representative of the rohe (region) where the isolate came from to support, speak to, and advise Māori contributing samples. These suggestions were echoed by young people, one stating

… in a modern medicine world, you just make an appointment and get a sample kind of thing. But to be more culturally appropriate there needs to be a real depth of understanding about why we take the long way around things sometimes and that’s just to protect ourselves and our wairua [spirit/soul].

Consent to taking and using TB isolates is a distinct but related matter. All participants felt that consent was important, especially at the time samples are collected. All groups felt that specific information about the collection of the sample/isolate should be provided to patients during the consent process, with groups specifying the variable level of detail they expected or hoped for—what would be done with their sample, what research it would be used to inform, the places it would move through, and the timeframe it would be stored and used. In a sense, the remarks above on tikanga and mauri also relate to informed consent, highlighting the importance of Māori contributors being informed of the implications of giving sputum samples in te ao Māori (Māori paradigms and environments).

Māori participants agreed that consent is not a one-off transaction. Consenting to a sputum sample being used in research must be specific, allowing for the permission of the contributor to cover a sample’s various movements and uses. This calls for staff collecting samples to take their time—to sit down and talk with contributors, communicate their intentions in detail, and answer their questions.

In contrast, participants in the Afghan focus groups expressed great ease with researchers and doctors deciding on the use of isolates. Their assumptions towards clinical process was expressed when asked about historical TB samples, where some Afghan participants assumed that people contributing those must have consented to their future use at the time. One Afghan participant emphasized that, as with any act that brings people comfort and well-being, research is culturally and religiously supported in their communities, suggesting their trust that research fosters well-being.

Perhaps the cultural variation in the small number of participants speaks to these two community’s distinct histories of clinical and state processes. Afghan participants spoke of having little experience with consent processes in Afghanistan and liking its relative prominence in NZ, whereas narratives from hui with Māori featured multiple stories of instances in which bodily substances and/or samples were taken without clear communication or consent. One kuia (female elder) recalled her whenua (placenta) being taken away “like rubbish” at childbirth and another related being told, after her hysterectomy, that the doctor without her knowledge or consent had also removed her appendix. These stories point to colonial aspects of clinical culture that have historically fostered distrust. Given the diversity of perspectives around consent, recommendations for best practice should not be based on an assimilationist, one-size-fits-all approach but should consider consent options based on self-identity, community values around sovereignty, and cultural safety.

### Personal Information and the Privacy of Samples

Participants were asked what kind of personal information ought to accompany research samples, both to inform the hypothetical study and in order for contributors to be contact traced in the future. The discussions this sparked highlight some of what is at stake for participants and their communities in contributing identifying information along with potential samples.

Every group appreciated the value of collecting contextualizing personal details for study, and generously offered ideas to what could be gathered: age, gender, cultural background, a bit of biography, general information about the person, place of birth, place of residence, and possibly school, suburb, mosque, NIH (National health Index) number (and associated information), hapū (sub-tribe), iwi (tribe), occupation, and information about social networks. A point stressed uniquely amongst Māori participants was that personal information worth collecting with an isolate sample would be contingent on the research question, and people contributing samples ought to be informed of the research aims. This position seems connected to the inalienability of isolate samples for Māori, as discussed in previous sections.

In every instance though, participants stressed that the necessity of confidentiality overrode any research benefits of attaching personal information to isolate samples. They were almost uniform in emphasizing that the anonymity of people who provide samples needs to be maintained through to dissemination. The stigmatizing social effects of being known to have TB at personal, familial, and community or iwi levels meant privacy was the only guarantee of protection and safety. People expressed real fear of exposure, suggesting in some discussions that it was an unthinkable outcome. “What would happen if the name was leaked?” the researchers asked one group and were told that “it could not happen; it would not be okay.” Being painted as a TB carrier can have long effects, with the whakamā (shame) passed down through generations.

A suggested operative principle for striking this balance between privacy and contextualizing information is that nobody should be traceable from information divulged. Two communities expressed a desire for their ethnic group, religious group, and/or national identity to be glossed in this study, too. One line of discussion amongst Pacific women explored the privacy-supporting implications of marking samples with an NIH number (as opposed to a name) against concern about having all the information attached to an NIH accompanying the sample.

Discussions of contact tracing were also framed by stigma-shaped anxieties. The Afghan participants noted that being cold-contacted in relation to TB could induce fear and shock about the level of access or surveillance of their personal information. For Afghan participants, and some Pacific participants, extreme care needed to be taken with contacting people who had had TB in the past.

### The Right to Speak For

A key challenge for retrospective consent processes involves balancing the need for privacy around TB status with the reliance on kin and community to care appropriately for old, unconsented samples. In many of the discussions, the scenario in which individuals could not be traced to give retrospective consent for their samples to be used was met with suggestions that groups such a family, hapū, iwi, or community groups could consent on their behalf. This was favoured in the discussion amongst kaumātua. There was a discussion that there should be a Pacific committee established to govern research decisions made over isolates from Pacific people, especially for historic samples.

Additionally, a charged conversation in a Pacific discussion suggested that the importance and impact of individual versus collective consent was not straightforward. When participants were asked if they had further questions at the end of the interview, this comment was made: “we are glad you are talking to us, as we think that we might want confidentiality around these isolates and what happens to them.” This research process raised the question of who is best to represent the community, which is particularly pertinent in communities who have distinct identities based on island, nation, religion within the broader group. One Pacific participant, acknowledging that they were participating in this study without having been elected by the broader community, noted that they would be filling the community in on this discussion. Two kuia in different groups also noted concern that a small number of kaumātua were conversing with this study’s researchers and that something this important ought to include a wider group. We can see from the discussion and raising of suggestions that each community trusts in science but only to a point. Beyond that point, as is variably determined, people tend to feel responsible for looking after their own. It is important enough that there is a form of thought-out collective governance on research in the community, but care and collective conversation is needed to determine the precise form of this, appropriate to the needs for privacy that are raised.

### TB, Love, and Collective Good

The importance of investment in collective good and collective well-being and the potential to realize this through TB research was an idea that recurred throughout all discussions. In spite of very real and valid concerns with stigma and traditional disempowering research processes, every group justified their support for TB research in terms of benefit to community: their immediate community, NZ society at large, and community-to-come (descendants and rangatahi). The Afghan interviewees expressed that this was a contribution they were very pleased to be asked to make. TB research, prevention, and tracing were tied to ideas of citizenship for Afghans, contributing to keeping NZ safe, and similarly, as acts of “love” for Pacific people. This way, notions of “love,” “aroha [love/compassion],” and acts of service were raised in almost all the discussions. Love was also raised as a guiding value with the potential to gently counter or even overcome TB stigma. For instance, “coming out” as having TB by getting tested, treated, and isolated was seen as an act of love to stop spreading infection.

## Discussion

When reviewing recommendations that participants offered in community discussions, a few things became apparent. Firstly, all groups called for more information and community education on TB. Secondly, the different groups’ feedback is generally compatible but with nuanced variations. For instance, they all raised informed consent as critical, but to diverse extents, and emphasized different points. Some proposed a model of flexible or shared data ownership, involving consent at the point of sample collection, and expressed ease with a scientific community making decisions over its future use. Others favoured the studied community holding sustained sovereignty of data samples. In this approach, the terms of consent delineate what specific questions one’s sample would be used to address and variations from this would require further discussion with the person who contributed that sample (or a whānau or iwi group that could speak for them). Meaningful guidelines should have the flexibility to speak to these nuances and to accommodate different communities’ tikanga.

One factor to consider is that “culturally specific” guidelines do not rigidly dominate clinical kaimahi (professionals)' interactions with people sharing isolate samples. Culturally informed guidelines can in best scenarios offer a frame for interactions around healthcare and research that dynamically inform the understanding and consent of all parties and are therefore effective and respectful. However, if treated as rigid and prescriptive, they can have the opposite effect. Writing on the limits of “cultural competence,” Curtis et al. (2019) suggest that culturally informed guidelines for practice can be detrimental when applied as a one-size-fits-all to any member of a particular group of people, for such usage assumes that people and culture are flat, unchanging, homogenous forms (which could not be farther from the truth). Here, Curtis et al. (2019) are speaking about “cultural competence” as a form of cross-cultural awareness that has been encouraged in clinicians and health professionals training and standards in NZ (and elsewhere). As well, guidelines that stipulate in detail how to treat a certain group of people may emphasize “acquiring knowledge, skills and attitudes … a ‘static’ level of achievement” (Curtis et al. 2019, 12). In contrast, guidelines based on cultural safety that embed reflective practices of health professional’s or researcher’s interpersonal power dynamics with patients or participants can result in more responsive and meaningful care for Indigenous people and other community groups. Therefore, rather than multicultural guidelines based in the clinical ethical system and cultural competency, we support the development of a set of guidelines that are culturally safe and based in Māori rights and sovereignty more broadly, yet recognize the diversity of ethnic groups in New Zealand.

Our suggestion here follows work that demonstrates the ethical value of Indigenous data sovereignty and data governance (Kukutai and Taylor 2016; Walter et al. 2021), particularly in health (Boulton et al. 2014). The key feature of such a move would be to “create value from” biological data, samples, and research involving Indigenous people that serves Indigenous rights and interests (Research Data Alliance International Indigenous Data Sovereignty Interest Group 2019). The interplay of individual and personal consent, as well as collective stewardship of isolate sample data, in responses to this study resonate with principles of kaitiakitanga (collective custodianship, guardianship). Kaitiakitanga is often used in relation to protecting and caring for the environment and natural resources. It also adapts well to health research contexts and underlying terms of Indigenous collection, custodianship (not ownership), and sharing of data (Boulton et al. 2014, 10). Hudson, Farrar, and McLean (2016) speak of experiences of Te Whakatōhea iwi in having an increasing stewardship over a range of datasets, health, and social records and knowledges in the rapidly evolving data context of NZ. The authors note that the different types of data call for different rights and interests (Hudson, Farrar, and McLean 2016, 172). For instance, Whakatōhea may hold exclusive rights over Indigenous knowledge, but rights are shared with government over service-level information created by iwi entities. Flexibility around custodianship and jurisdiction of data need to be responsive to the diverse meanings that custodianship holds for different communities. Within custodianship, guidelines need to consider who should be responsible for it and whether it should it be shared or involve partial custodianship of samples. As not all communities were comfortable with research or health policy decisions being made about the samples they donated without follow-up consent, community governed or partnered options could be considered in future guidelines. For Māori data sovereignty, Kukutai et al. (2023) advocate for iwi and hapū data to be under the control and governance of iwi and hapū through tika (safe) processes that benefit Māori. Under this Indigenous collective framework where safety and benefit are priorities, the processes themselves—rather than individual or collective tensions—would determine responsibility for samples. This level of ethical governance would require the decolonization of current data systems (Kukutai and Cormack 2020) to dismantle “the structures that perpetuate the dispossession of Māori and Māori data, while shifting the locus of control over Māori data back to Māori” (Kukutai et al. 2023, 18).

Responses from participants in this study raise the question of what it means to be citizens and particularly *good* citizens, in a country with tangata whenua (Indigenous people) who are guaranteed *good* governance through rights of self-determination and governance under national and local treaties and declarations (Reid 2013). The capacity of immigrant groups to realize “good citizenship” is limited and heavily affected by government policies and paradigms determining whether and how they could be citizens at all. These material and relational conditions compound with gaps in information about TB transmissibility and treatment and with social and symbolic forces to shape people’s anxieties. Giving credence to these worries, Badu, Mpofu, and Farvid (2018a, b) relate health workers’ concerns over unclear and inconsistent application of New Zealand’s immigration rules for people who have records of having TB, with many visa applicants citing drug resistance to TB being denied. They suggest these patterns feed a social anxiety and secrecy around TB—people on migrant visas may prioritize their immigration status over their health and avoid seeking diagnosis and treatment. Addressing such insecurities by removing the discriminatory clause around TB in people’s visa application processes would arguably do a lot to support such communities in moving forward from stigmatizing narratives around TB, as well as benefiting efforts to control and treat cases of TB.

This example and suggestion show how important it is that ethical governance at the micro level—labs, clinics, governing bodies—is supported by a broader ethical redress. Ideally, in a practice of cultural pluralism under a properly bicultural government structure, in which new migrants are positioned in relation to sovereign Māori (rather than this relation being mediated heavily by the state, as it currently is), it is possible these material insecurities and concerns that augment some participants’ worries and fears could be allayed—though even the current nation state of NZ could be recognized as in relation to and in reciprocity with other island nations in the Pacific. As Park et al. (2011) note in their application of the social theory of whakapapa to an ethical and morally nuanced policy development,

New Zealand could use the whakapapa model to find ways to reciprocate for the benefits received and harms done. The principle of utu [reciprocation] would support being more hospitable to people … who want—or seriously need—to include New Zealand as part of their transational lives. (23)

Consistent across all the discussions was the stigma of TB and people’s interest in lessening this. It is worth underscoring the hope that participants expressed in collectively growing beyond stigma and also the unanimous interest in doing what benefits collective well-being. This informed most people’s ultimate support of TB research. People’s material security is critical to their capacity to enact such community-oriented forms of aroha. Fiona Cram (2020) demonstrates as much in her analysis of Māori-led initiatives to distribute food packages, hygiene packages, and sociality during NZs stringent COVID-19 lockdown. These efforts mitigated whānau vulnerability to exacerbated social and financial hardship in that time. Cram notes that, anecdotally, Māori who were most able to do such service work or mahi aroha were those in secure rentals or who owned their own homes. Cram’s argument speaks powerfully to how critical material comfort, security, and support are to realizing collectivizing efforts, such as those that would inform people’s comfortable participation in, and governance of, TB research.

Although this research was able to capture some community perceptions of culturally appropriate approaches to human-derived microbial-pathogen laboratory research, as a small scoping study, it was not without limitations. As the project only worked with a relatively small number of people across three broad ethnic groups, the findings cannot be generalized within or across communities who experience TB. Further research with more participants and more communities—particularly Asian ethnic groups (who inequitably experience the highest rates of TB in NZ)—is needed. Furthermore, as the study has translated much of the qualitative data from diverse languages into English, some of the deeper meaning of the data may not have been fully captured in this process. Despite these limitations, the research does provide a foundational platform to elicit discussions and promote further research in this area.

This paper’s findings support the urgent need to develop culturally responsive ethical guidelines for the collection, storage, use, and governance of pathogen isolates in New Zealand that are predicated on Indigenous rights, yet acknowledge cultural pluralism. Furthermore, the research has demonstrated the merits of undertaking robust and dynamic consultative processes with communities to inform such guidelines and ensure sustainable and meaningful Indigenous sovereignty, and safe, responsive practices for all communities who contribute their pathogen isolates for research. As encapsulated by a prominent Māori proverb (from the iwi Ngāti Kahungunu), “ēhara tāku toa i te toa takatahi, engari he toa takitini”—our strength is not made from us alone but made from many.

## Data Availability

Given the qualitative nature of the study and ethic requirements, data is not available to share from this research.
